# Pericoronary Adipose Tissue Radiomic Features and Quantitative Plaque Analysis in Coronary Artery Disease: Insights from Coronary Computed Tomography Angiography

**DOI:** 10.3390/diagnostics16081174

**Published:** 2026-04-15

**Authors:** Konstantin V. Zavadovsky, Alexey V. Kalinovsky, Alina N. Maltseva, Kristina V. Kopeva, Olga V. Mochula, Ayana S. Dasheeva, Andrew V. Mochula, Elena V. Grakova

**Affiliations:** 1 Department of Radiology and Tomography, Cardiology Research Institute, Tomsk National Research Medical Center, Russian Academy of Sciences, Tomsk 634012, Russia; kalinovskiyalex169@mail.ru (A.V.K.); maltseva.alina.93@gmail.com (A.N.M.); mochula.olga@gmail.com (O.V.M.); dasheevayana@gmail.com (A.S.D.); 2 Department of Ambulatory Cardiology, Cardiology Research Institute, Tomsk National Research Medical Center, Russian Academy of Sciences, Tomsk 634012, Russia; kristin-kop@inbox.ru (K.V.K.); gev@cardio-tomsk.ru (E.V.G.); 3 Nuclear Department, Cardiology Research Institute, Tomsk National Research Medical Center, Russian Academy of Sciences, Tomsk 634012, Russia; mochula.andrew@gmail.com

**Keywords:** coronary computed tomography angiography, coronary artery disease, pericoronary adipose tissue, radiomics, coronary atherosclerosis

## Abstract

**Background/Objectives:** Coronary computed tomography angiography (CCTA) is a modern method for assessing the total burden of atherosclerotic lesions. The perivascular fat attenuation index (PFAI) is a reliable predictor of major adverse cardiovascular events (MACE). Radiomics extracts substantially more information from images than visual assessment by radiologists. However, the relationships between quantitative parameters of coronary atherosclerosis, the PFAI, and radiomic features of pericoronary adipose tissue (PCAT) in patients with coronary artery disease (CAD) remain unclear. The study aimed to evaluate the associations between PCAT characteristics, including radiomic features, and quantitative parameters of coronary atherosclerosis in stable CAD patients. **Methods:** The study included 79 patients with stable CAD who underwent CCTA. The patients were divided into two groups: nonobstructive CAD (NOCAD, stenosis < 50%; *n* = 61) and obstructive CAD (OCAD, stenosis ≥ 50%; *n* = 18). The CCTA data were analyzed to quantify coronary atherosclerosis parameters (plaque volume and burden), the PFAI, PCAT volume, and radiomic features of PCAT in the proximal segments of major coronary arteries. **Results:** The study included 79 patients: NOCAD group = 61 patients (age 57.00 (50.00–65.00) years) and OCAD group = 18 patients (age 60.5 (55.75–65.75) years). The OCAD patients exhibited higher plaque volume and burden across all components. No significant between-group differences were observed in PFAI or PCAT volume for any vessel. However, 50% (46/92) of PCAT radiomic features in the proximal right coronary artery (RCA) differed significantly between groups, 42 of which were textural. The PFAI correlated most strongly with soft tissue (ST) plaque volume (ρ = −0.22), and burden (ρ = −0.21) of the soft tissue component of plaques (*p* < 0.001). The PCAT volume significantly correlated (*p* < 0.001) with plaque volume (ρ = 0.30) and with individual components—soft tissue (ρ = 0.30), fibrous–fatty (ρ = 0.27), fibrous (ρ = 0.30), calcified (ρ = 0.22), and non-calcified (ρ = 0.30)—as well as with the burden of the soft tissue component (ρ = 0.26). **Conclusions:** The radiomic features of RCA PCAT differed significantly between the NOCAD and OCAD groups. Quantitative coronary atherosclerosis parameters showed significant associations with the PCAT radiomic features in CAD patients, potentially serving as independent predictors of the MACE risk. In contrast, the PFAI values did not differ between groups and neither PFAI nor PCAT volume associated with atherosclerosis parameters.

## 1. Introduction

Cardiovascular diseases remains the leading cause of death worldwide, with coronary artery disease (CAD) accounting for approximately 13% of deaths in women and 14% of those in men [1].

Coronary computed tomography angiography (CCTA) has emerged as a robust noninvasive modality for the evaluation of coronary atherosclerosis in CAD patients. Quantitative CCTA enables comprehensive plaque characterization, including the assessment of coronary lumen volume and total plaque burden, with differentiation of non-calcified (lipid-rich and fibrous–fatty), fibrous, and calcified components [2,3].

Coronary atherosclerosis is characterized by vascular inflammation and plaque instability, which may lead to plaque erosion or rupture. Inflammation within the vessel wall is associated with changes in pericoronary adipose tissue (PCAT) attenuation, quantified by the fat attenuation index (FAI). Increased FAI reflects edema and inflammatory cell infiltration in adjacent adipose tissue and may serve as an indirect marker of the intensity of coronary inflammation [4]. FAI, defined as the mean X-ray attenuation of PCAT within a predefined region of interest on coronary computed tomography angiography, has been reported as an independent predictor of major adverse cardiovascular events (MACEs) [5].

Over the past decade, advances in image post-processing have expanded the analytical capabilities of cardiovascular imaging. Technologies such as radiomics enable the extraction of considerably more information from medical images than can be obtained through visual analysis by a radiologist [6,7,8]. This approach supports disease phenotyping and characterization of pathological changes through statistical descriptors derived from individual voxels and their spatial relationships, reflecting underlying tissue properties. Radiomic features capture subtle variations in voxel intensity and inter-voxel heterogeneity that may not be apparent on routine qualitative evaluation.

However, the relationship between quantitative CCTA-derived plaque metrics and detailed radiomic PCAT feature in patients with obstructive CAD (OCAD) and nonobstructive CAD (NOCAD) remains insufficiently characterized. Despite the well-known prognostic utility of PFAI and the application of PCAT radiomics to predict plaque progression, it remains unclear whether PCAT radiomic phenotypes reflect the quantitative plaque compositional burden and discriminate OCAD from NOCAD more effectively than conventional attenuation and volume variables [5,9,10,11,12,13]. Establishing associations between PCAT radiomic features, quantitative measurements of coronary atherosclerosis and PCAT in NOCAD and OCAD patients may improve risk stratification for cardiovascular complications and enable a more refined quantitative assessment of pericoronary inflammation associated with atherosclerosis.

The study aimed to evaluate the associations between PCAT characteristics, including radiomic features, and quantitative measures of coronary atherosclerosis in stable CAD patients.

## 2. Materials and Methods

### 2.1. Patients and Design

The study included 79 patients with stable CAD who underwent CCTA between 2019 and 2025. All examinations were performed in accordance with contemporary clinical guidelines [14,15]. The present study was an observational investigation.

Based on the extent of coronary atherosclerosis and the degree of luminal stenosis, the patients were classified into two groups: NOCAD (stenosis < 50%; *n* = 61) and OCAD (stenosis ≥ 50%; *n* = 18). Within the OCAD population, there were 13 (72%) patients with single-vessel disease, 3 (17%) with double-vessel disease, and 2 (11%) with triple-vessel disease.

The inclusion criteria were CAD confirmed by CCTA, high-quality CT images suitable for visual and quantitative assessment of coronary vessels, left ventricular (LV) ejection fraction ≥ 50%, a regular sinus rhythm, and written informed consent to participate in the study. The exclusion criteria were LV hypertrophy or dilation on CCTA; prior myocardial infarction or revascularization; uncontrolled or resistant arterial hypertension; decompensated diabetes mellitus; morbid obesity (body mass index (BMI) ≥ 40 kg/m^2^); chronic kidney disease of stage > 2; moderate or severe valvular stenosis and/or regurgitation; cardiomyopathies; ventricular extrasystoles of Lown grade ≥ 3; second- or third-degree atrioventricular block or sick sinus syndrome; acute or chronic inflammatory heart disease; severe bronchial asthma and/or chronic obstructive pulmonary disease; systemic inflammatory disease; and active malignant neoplasms.

The research protocol was approved by the Local Ethical Committee of Cardiology Research Institute, Tomsk National Research Medical Center, Russian Academy of Sciences (protocol No. 10, dated 18 February 2021), and all participants provided signed informed consent. The study complied with the Declaration of Helsinki guidelines for all human research.

A total of 237 coronary vessels—left anterior descending artery (LAD), left circumflex artery (LCX), and right coronary artery (RCA) of each patient were analyzed. Of these, 213 (89.9%) had nonobstructive lesions and 24 (10.1%) had obstructive lesions.

CCTA was used to quantify the anatomical degree of luminal stenosis (%) in the left main coronary artery (LMA) and each of the three major epicardial coronary arteries (LAD, LCX, RCA) to classify patients as having NOCAD and OCAD (>50% stenosis in ≥1 coronary artery). Quantitative measures of coronary atherosclerosis, including total plaque volume and plaque burden, pericoronary adipose tissue (PCAT) attenuation parameters (pericoronary fat attenuation index) surrounding proximal vessel segments, and radiomic features of PCAT derived from segmented proximal vessel images were evaluated.

### 2.2. Coronary Computed Tomography Angiography

CCTA examinations were performed using standardized protocol on multiple CT systems (Revolution EVO 64, GE Healthcare, Milwaukee, WI, USA; Discovery NM/CT 570c hybrid system, GE Healthcare, Milwaukee, WI, USA). Patient preparation, study protocol, image acquisition, reconstruction, and analysis have been described previously [16,17].

Contrast enhancement was achieved via intravenous administration of 80–90 mL iopromide (Ultravist 370 mg I/mL; Bayer, Leverkusen, Germany) at a flow rate of 5 mL/s. The estimated effective radiation doses ranged from 1.5 to 3.0 mSv with prospective ECG synchronization and 15–19 mSv with retrospective ECG synchronization acquisition.

### 2.3. Quantitative Analysis of Atherosclerosis Based on CCTA Data

CCTA images were reconstructed at 75% of the cardiac cycle using standard reconstruction kernel, with a slice thickness of 0.625 mm and an increment of 0.625 mm. In cases of rhythm-related artifacts, additional reconstructions were performed, including phase 40% of the cardiac cycle. All reconstructed images were transferred to a post-processing workstation (Advantage Workstation VolumeShare 7; GE Healthcare, Milwaukee, WI, USA). Image post-processing was performed at the Core Facility “Medical Genomics”.

Coronary atherosclerosis analysis was restricted to coronary segments free of significant artifacts. The degree of stenosis was assessed on curved and stretched multiplanar reconstructions, as well as cross-sectional images using dedicated software (CardIQ Xpress 2.0 Reveal, GE Healthcare, Milwaukee, WI, USA). Manual correction of the vessel centerline and luminal contour was performed as necessary. No significant artifacts impeding analysis were observed. Short arteries (e.g., left main artery and diagonal arteries) or those with a diameter < 1.5 mm were excluded from processing.

Atherosclerotic plaque components were defined as follows: soft tissue component, with a range of values from −30 to 30 Hounsfield units (HUs); fibrous–fatty component, with a range of values from 31 to 130 HUs; fibrous component, with a range of values from 131 to 350 HUs; calcified component, with values greater than 350 HUs [18]. The overall quantitative parameters of coronary atherosclerosis were then determined as follows:

• TLV—total lumen volume, mm^3^;
• TPV—total plaque volume, mm^3^;
• TPV-ST—total plaque volume of the soft tissue component, mm^3^;
• TPV-FF—total plaque volume of the fibrous–fatty component, mm^3^;
• TPV-F—total plaque volume of the fibrous component, mm^3^;
• TPV-C—total plaque volume of the calcified component, mm^3^;
• TPV-NC—total plaque volume of the non-calcified component, mm^3^;
• TPB—total plaque burden, %;
• TPB-ST—total plaque burden of the soft tissue component, %;
• TPB-FF—total plaque burden of the fibrous–fatty component, %;
• TPB-F—total plaque burden of the fibrous component, %;
• TPB-C—total plaque burden of the calcified component, %;
• TPB-NC—total plaque burden of the non-calcified component, %.

The LMA and diagonal branches were considered as part of the LAD; the LCX included obtuse marginal branches, ramus intermedius, and left posterior lateral and left posterior descending arteries (in left-dominant coronary circulation); the right posterior lateral and right posterior descending arteries were included in the RCA (in right-dominant circulation). Lateral branches (diagonal, obtuse marginal, posterior descending, and posterior lateral) were analyzed if sufficiently visualized. For vessels with multiple branches, the parameters were summed. Total plaque volumes were calculated by summing volumes across coronary arteries (LAD, LCX, and RCA). Quantitative coronary atherosclerosis analysis followed the current QCCTA guidelines [19].

### 2.4. Quantitative Analysis of PCAT Based on CCTA Data

The volume and attenuation of PCAT were assessed on ECG-synchronized post-contrast images reconstructed with a slice thickness of 0.625 mm using the processing workstation (Advanced Workstation, GE Healthcare, Milwaukee, WI, USA). The Auto Coronary Analysis software (v. 4.7) module was used for segmentation. Regions of interest were delineated around the major coronary arteries using the Brush tool on stretched multiplanar reconstructions. For the right coronary artery (RCA), PCAT was evaluated along a 10–50 mm proximal segment, starting 10 mm distal to the ostium, whereas for the left anterior descending (LAD) and left circumflex (LCX) arteries, a 40 mm proximal segment was analyzed [20]. The radial extent of PCAT was defined as a distance from the outer coronary vessel wall equal to one local luminal diameter. Manual adjustments were applied when necessary to ensure accurate delineation and segmentation. Within each region of interest, the PCAT volume and the pericoronary fat attenuation index (PFAI) were quantified. The PFAI was defined as the mean CT attenuation of PCAT within the predefined region of interest. The volumetric regions of interest were exported in DICOM format for subsequent radiomic analysis.

### 2.5. Radiomic Analysis of PCAT

Radiomic analysis was performed using the dedicated software (3D Slicer v. 5.7.0, The Slicer Community, Boston, MA, USA). Segmented DICOM images of the PCAT surrounding the major coronary arteries—LAD, LCX, and RCA—were imported into 3D Slicer and processed with the Segment Editor module. Within each region of interest, voxels with attenuation values between −190 and −30 HUs were selected using the Threshold tool (v. 4.7).

For each coronary segment, 92 radiomic features were extracted using the PyRadiomics package (v. 842cdf 2024-11-13). These included 17 first-order features and 75 texture features: 24 gray-level co-occurrence matrix (GLCM), 14 gray-level dependence matrix (GLDM), 16 gray-level run length matrix (GLRLM), 16 gray-level size zone matrix (GLSZM), and 5 neighborhood gray-tone difference matrix (NGTDM) parameters. Prior to feature extraction, voxel intensities were discretized into 25 bins of equal width (bin width, 6.4 HUs) to reduce image noise while preserving sufficient resolution to capture biologically relevant spatial variations in PCAT attenuation.

### 2.6. Statistical Analysis

Statistical analysis and graphical images were performed/created using the statistical software R, version 4.5.0 (R Foundation for Statistical Computing, Vienna, Austria). Normality was assessed using the Shapiro–Wilk test. Continuous variables showing a normal distribution were expressed as mean ± standard deviation, whereas non-normally distributed variables are presented as the median with interquartile ranges. Between-group comparisons of independent samples were performed using the Mann–Whitney U test. The associations between continuous variables were evaluated using Spearman rank correlation analysis. Given the exploratory nature of these analyses and the large number of tested variables, no formal correction for multiple comparisons (e.g., Bonferroni adjustment) was applied. Post hoc power analysis demonstrated that the achieved statistical power for the primary between-group comparisons ranged from 0.8 to 0.9, indicating that the sample size was sufficient to detect clinically meaningful differences. All statistical tests were two-sided, and statistical significance was defined as *p* < 0.05.

## 3. Results

The study included 79 patients of whom 47 were men (59.5%). Sixty-one patients were classified as having NOCAD and 18 as having OCAD. To address scanner heterogeneity, the patients were well-balanced across CT systems within groups: NOCAD (*n* = 28 (46%) Revolution EVO 64; *n* = 33 (54%) Discovery NM/CT 570c hybrid system) and OCAD (*n* = 10 (56%) Revolution EVO 64; *n* = 8 (44%) Discovery NM/CT 570c hybrid system).

Clinical and demographic characteristics are presented in Table 1. Compared with the NOCAD group, the patients with OCAD had a higher prevalence of dyslipidemia, obesity, and diabetes mellitus (all *p* < 0.05).

A comparative analysis of quantitative atherosclerosis parameters and PCAT between the groups is presented in Table 2.

In our prior study, we demonstrated good (0.75 < ICC < 0.90) to excellent (ICC > 0.90) intra- and inter-observer reproducibility for coronary atherosclerosis volume and burden parameters [16].

**Figure 1:**
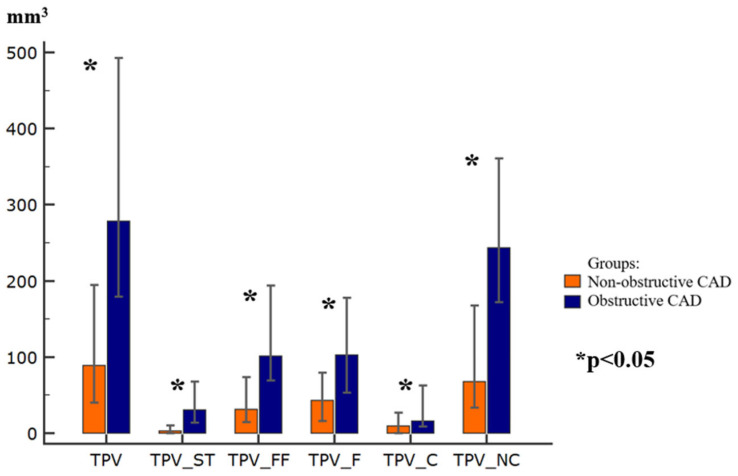
Differences in plaque volume parameters. Notes: TPV—total plaque volume; TPV_ST—total plaque volume of the soft tissue component; TPV_FF—total plaque volume of the fibrous–fatty component; TPV_F—total plaque volume of the fibrous component; TPV_C—total plaque volume of the calcified component; TPV_NC—total plaque volume of the non-calcified component; CAD—coronary artery disease.

**Figure 2:**
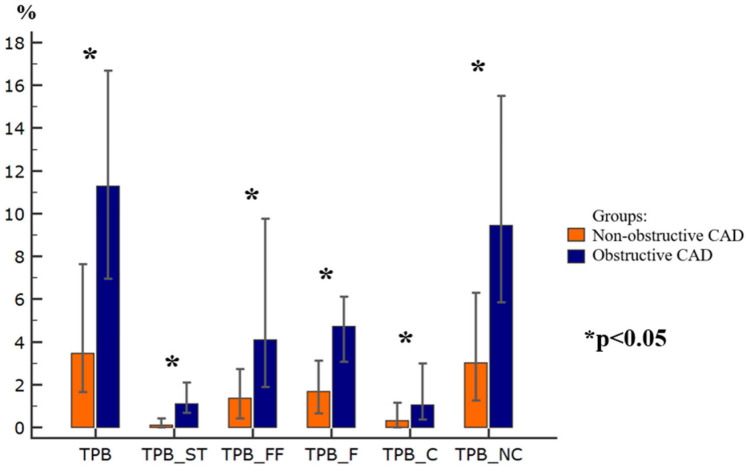
Differences in plaque burden parameters. TPB—total plaque burden; TPB_ST—total plaque burden of the soft tissue component; TPB_FF—total plaque burden of the fibrous–fatty component; TPB_F—total plaque burden of fibrous component; TPB_C—total plaque burden of the calcified component; TPB_NC—total plaque burden of the non-calcified component; CAD—coronary artery disease.

Compared to the NOCAD group, the patients with OCAD demonstrated significantly higher quantitative measures of coronary atherosclerosis (all *p* < 0.05). Specifically, TPV, TPV-ST, TPV-FF, TPV-F, TPV-C, TPV-NC, TPB, TPB-ST, TPB-FF, TPB-F, TPB-C, and TPB-NC were increased in the OCAD group. In contrast, total lumen volume (TLV) did not differ significantly between the groups. Figure 1 and Figure 2 present a comparative analysis of these coronary atherosclerosis parameters.

No statistically significant intergroup differences were observed in the quantitative PCAT parameters (PFAI and PCAT volume) across the LAD, LCX, and RCA arteries.

**Figure 3:**
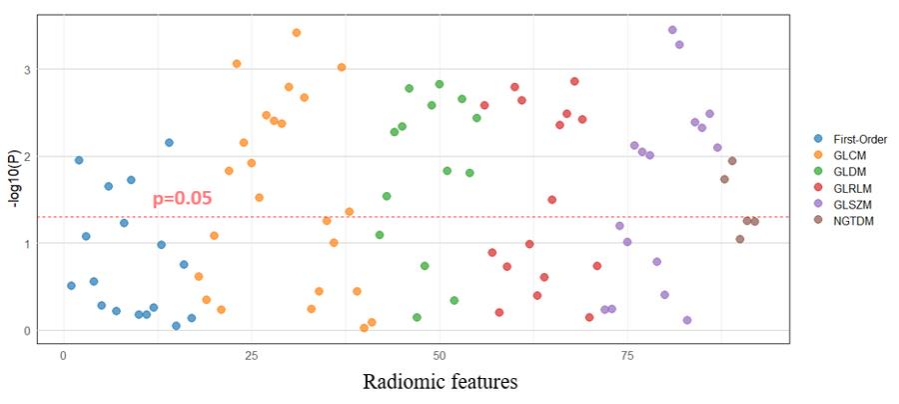
Manhattan plot of radiomic features derived from right coronary artery pericoronary adipose tissue. Notes: Manhattan plot showing the −log10(*p*) values for radiomic features extracted from right coronary artery (RCA) pericoronary adipose tissue (PCAT), comparing obstructive and nonobstructive coronary artery disease groups; the horizontal dashed line indicates the significance threshold (*p* = 0.05); features above the line are statistically significant; radiomic feature classes include first-order statistics, gray-level co-occurrence matrix (GLCM), gray-level dependence matrix (GLDM), gray-level run length matrix (GLRLM), gray-level size zone matrix (GLSZM), and neighborhood gray-tone difference matrix (NGTDM).

No significant differences were observed between the NOCAD and OCAD groups in quantitative PCAT parameters, including the FAI and the PCAT volume, across LAD, LCX, and RCA. In contrast, several radiomic features derived from RCA PCAT differed significantly between patients with obstructive and nonobstructive lesions. These associations are illustrated in the Manhattan plot (Figure 3).

Significant differences between the groups were identified in 46 of 92 (50%) radiomic features derived from right coronary artery (RCA) pericoronary adipose tissue (PCAT). These included features from first-order, GLCM, GLDM, GLRLM, GLSZM, and NGTDM classes (Supplementary Table S1).

Table 3 presents Spearman’s correlation coefficients (ρ) for statistically significant associations between quantitative parameters of coronary atherosclerosis and PCAT metrics across the evaluated vessels across.

**Figure 4:**
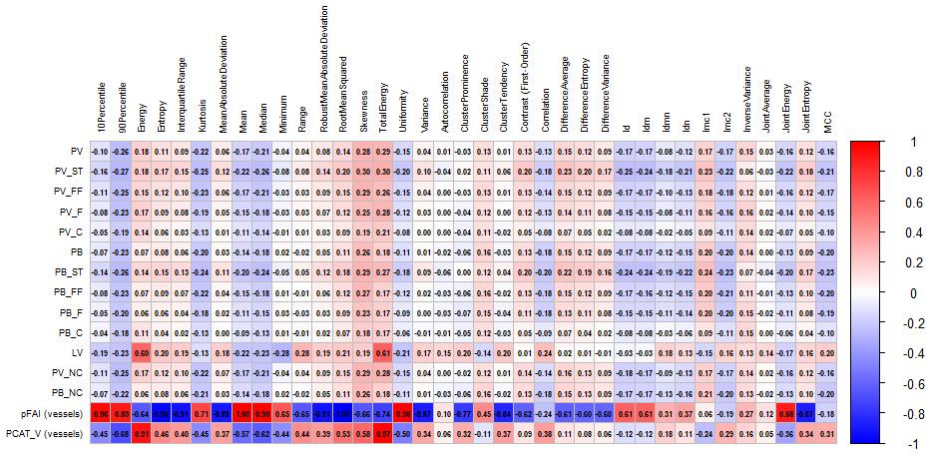
Heat map of spearman correlation coefficients between coronary atherosclerosis parameters and PCAT radiomic features (Part 1). Heat map illustrating Spearman correlation coefficients (ρ) between quantitative coronary atherosclerosis parameters and radiomic features derived from pericoronary adipose tissue (PCAT). Warmer colors indicate positive correlations, and cooler colors indicate negative correlations. Correlation strength is displayed on a continuous scale from −1 to 1.

**Figure 5:**
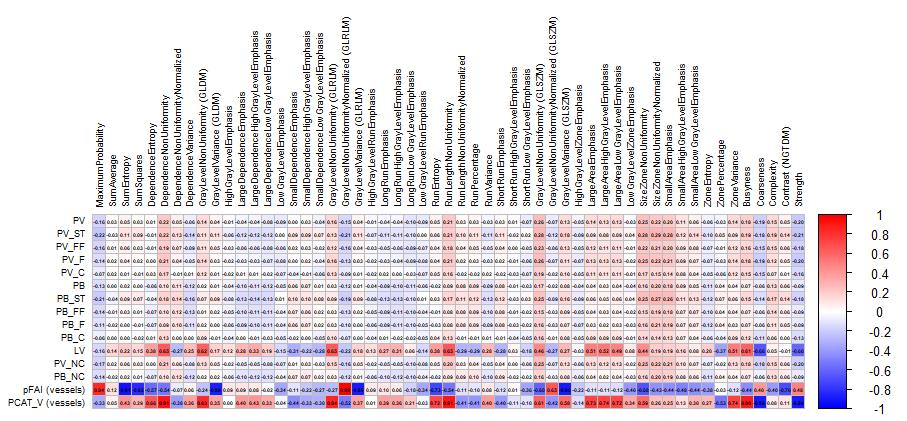
Heat map of spearman correlation coefficients between coronary atherosclerosis parameters and PCAT Radiomic Features (Part 2). Notes. Continuation of the heat map analysis showing additional radiomic features. Spearman correlation coefficients (ρ) are color-coded according to magnitude and direction, with values ranging from −1 to 1. Abbreviations: PV—plaque volume; ST—soft tissue component; FF—fibrous–fatty component; F—fibrous component; C—calcified component; NC—non-calcified component; PB—plaque burden; PFAI—perivascular fat attenuation index; PCAT_V—pericoronary adipose tissue volume.

Quantitative PCAT parameters demonstrated significant weak to moderate negative correlations for the FAI and positive correlations of the PCAT volume with measures of coronary atherosclerosis. The soft tissue plaque component, expressed as both volume and burden, exhibited the strongest inverse correlations with vessel FAI. The PCAT volume correlated positively with TPV, as well as with the soft tissue, fibro-fatty, fibrous, calcified, and non-calcified plaque components and the soft tissue plaque burden. In addition, coronary lumen volume showed an inverse correlation with the FAI and a direct correlation with the PCAT volume. Significant associations were also observed between PCAT radiomic features and plaque volume and burden, as well as between PCAT density and volume. The magnitude and direction of these correlations are illustrated in heat maps (Figure 4 and Figure 5).

## 4. Discussion

According to CCTA, all quantitative measures of coronary atherosclerosis, including plaque volumes (TPV, TPV-ST, TPV-FF, TPV-F, TPV-C, and TPV-NC), as well as plaque burdens (TPB, TPB-ST, TPB-FF, TPB-F, TPB-C, and TPB-NC) were higher in the OCAD patients than in those with NOCAD. Notably, the differences between the groups were driven predominantly by the soft tissue plaque component.

### 4.1. Quantitative Plaque Volume and Burden: Prognostic Value in NOCAD and OCAD Patients

Contemporary investigations of quantitative coronary atherosclerosis measures focus on their diagnostic and prognostic implications, particularly in relation to adverse cardiovascular events. In the SCOT-HEART trial, Williams et al. demonstrated that the burden of low-attenuation plaque (<30 HU) was associated with an increased risk of fatal or nonfatal acute myocardial infarction (AMI) in patients with both NOCAD and OCAD [21]. It was found that a low-density (soft tissue) plaque burden >4% was identified as an independent predictor of subsequent fatal or nonfatal AMI in NOCAD and OCAD patients. Our findings support the concept that quantitative CCTA-derived assessment of atherosclerosis may contribute to risk stratification for AMI. Volumetric plaque parameters, particularly those reflecting the low-attenuation components, may provide incremental information in patients with both NOCAD and OCAD. In the present study, soft tissue plaque volume was significantly higher in patients with obstructive atherosclerosis than in those with nonobstructive lesions (median 30.5 vs. 2.6 mm^3^), suggesting a more adverse plaque phenotype in the obstructive group. These observations are consistent with prior evidence linking increased low-attenuation plaque burden to a higher risk of subsequent AMI and may indicate greater vulnerability in this population [21].

### 4.2. Pericoronary Adipose Tissue Radiomic Features in NOCAD and OCAD Patients

Despite the absence of significant differences in PCAT density and volume between the groups, radiomic analysis of RCA PCAT identified significant intergroup differences in half of the extracted features. These findings suggest that PCAT radiomic features may capture aspects of tissue remodeling not reflected by conventional metrics such as the fat attenuation index (FAI) or the PCAT volume. Prior studies have demonstrated associations between PCAT radiomic signatures and vascular inflammation and adverse plaque characteristics [4,9,10]. In this context, the radiomic profile may provide more information regarding the inflammatory and structural heterogeneity of PCAT in both NOCAD and OCAD compared to PCAT attenuation and volume. The groups differed by texture features (GLCM, GLDM, GLRLM, GLSZM, and NGTDM), which describe the spatial relationships between voxel characteristics, and thus consider the inflammatory phenotype of PCAT and the heterogeneity of pathophysiological processes.

Weak but statistically significant negative correlations were found between the quantitative measures of coronary atherosclerosis and the PFAI. This finding differs from prior reports, demonstrating a positive association between increasing plaque burden and higher (less negative) PCAT attenuation values [2]. Although the coronary artery calcium score is a valuable prognostic tool, it does not reflect the presence of non-calcified plaques or the degree of vascular inflammation. In contrast, the FAI obtained from CCTA serves as a surrogate marker of coronary inflammation and has demonstrated predictive value for adverse cardiac outcomes [22]. The discrepancy between our findings and previous data may be attributable to the predominance of the NOCAD patients in the present cohort. In this subgroup, lower-grade or more diffuse inflammatory activity may modify the relationship between plaque burden and the FAI compared with the patients with OCAD. These observations suggest that the association between coronary atherosclerosis and PCAT attenuation may vary according to disease phenotype and inflammatory intensity. Some studies have demonstrated that increased PCAT attenuation on CT robustly predicts cardiovascular risk and local inflammation in stable patients [11,23]; however, more recent investigations report inconsistent associations with long-term adverse events after adjustment for stenosis severity and metabolic confounders, underscoring heterogeneity in PCAT prognostic utility across cohorts [24,25]. The inverse correlations observed between the PFAI and plaque metrics in our study should be interpreted in light of emerging evidence that the relationship between RCAT and coronary atherosclerosis is composition-dependent rather than purely burden-dependent. Guo et al. (2025) reported that while the FAI values were higher in vessels with vulnerable plaque phenotypes, total plaque burden showed an inverse association with the FAI in multivariable analysis, suggesting a non-monotonic relationship [26]. Similarly, Jing et al. (2024) demonstrated that the periplaque FAI was positively associated with lipid plaque fraction but negatively associated with calcified plaque fraction [27]. Zhang et al. (2025) further showed that increases in the FAI were driven primarily by necrotic core components, whereas fibrous plaque components exerted an opposing effect [28]. Studies on acute coronary syndrome populations have used lesion-specific FAI measurements and demonstrated strong associations with vulnerable plaque characteristics, which may not be directly comparable to patient-level or proximal-segment measurements in stable CAD cohorts [29].

In the present study, PCAT volume was positively associated with quantitative measures of the coronary atherosclerosis burden. To our knowledge, no prior publications have specifically evaluated PCAT volume within a predefined region of interest. The observed association may reflect two complementary mechanisms. First, progressive atherosclerosis may lead to an increase in vessel diameter through positive remodeling mechanism, thereby expanding the anatomical region of interest and the measurable PCAT volume. Second, increasing plaque burden may be accompanied by a more extensive inflammatory response within PCAT, resulting in greater infiltration of the vessel wall and adjacent adipose tissue.

Significant correlations were observed between quantitative coronary atherosclerosis measures and radiomic features of PCAT. These findings support the potential role of PCAT radiomic phenotyping in improving risk stratification among stable CAD patients.

Future investigations should extend this approach to epicardial adipose tissue (EAT), given prior evidence that EAT radiomic phenotypes are associated with the risk of major adverse cardiovascular events (MACEs) [30]. Integrating PCAT and EAT radiomic characterization may enhance the prediction of adverse outcomes in patients with CAD.

## 5. Limitations

This study has several limitations. This single-center observational study design, relatively small study sample, an imbalanced distribution between the NOCAD and OCAD groups may limit statistical power and generalizability, as well as increase the risk of type II error. Multiple radiomic features were analyzed without external validation, increasing the risk of overfitting. Variability related to CT acquisition parameters and segmentation methodology may also have influenced radiomic measurements. Furthermore, radiomic analysis was limited to RCA PCAT, as this segment is best described in the literature. We recognize that vessel-level or plaque-level analyses may offer more detailed insights into local inflammatory processes and should be explored in future investigations. Therefore, these results should be regarded as preliminary and hypothesis-generating. Future multicenter investigations involving more diverse patient cohorts are warranted to confirm and extend these observations.

## 6. Conclusions

In this study, quantitative CCTA-derived measures of coronary atherosclerosis showed significant associations with radiomic features of pericoronary adipose tissue (PCAT) in NOCAD and OCAD patients, although the correlation coefficients were weak. Conventional PCAT metrics, including density and volume, showed no significant intergroup differences and were not associated with other imaging parameters.

Radiomic features of PCAT, predominantly texture-based metrics, differed between obstructive and nonobstructive lesions and were strongly associated with the atherosclerosis burden. These findings suggest that selected PCAT radiomic features might be considered as promising imaging biomarkers of pericoronary inflammation and that they require future investigation to elucidate their potential contribution to risk stratification for cardiovascular complications in NOCAD and OCAD patients.

## Figures and Tables

**Table 1:** Clinical and demographic characteristics of NOCAD and OCAD patients.

Parameters	All Patients (*n* = 79)	NOCAD (*n* = 61)	OCAD (*n* = 18)	*p*-Value
Male sex, *n* (%)	47 (59.49)	34 (55.74)	13 (72.22)	0.21
Age, years	58.00 (52.00; 65.00)	57.00 (50.00; 65.00)	60.50 (55.75; 65.75)	0.33
BMI, kg/m^2^	28.82 ± 4.15	29.15 ± 4.25	27.71 ± 3.71	0.20
Angina, *n* (%)	71 (89.87)	56 (91.80)	15 (83.33)	0.30
Dispnoe, *n* (%)	54 (68.35)	45 (73.77)	9 (50.00)	0.06
Hypertension, *n* (%)	71 (89.87)	54 (88.52)	17 (94.44)	0.46
**Dislipidemia, ** ***n*** ** (%)**	**50 (63.29)**	**34 (55.74)**	**16 (88.88)**	**0.01**
**Obesity, ** ***n*** ** (%)**	**40 (50.63)**	**26 (42.62)**	**14 (77.77)**	**0.01**
Current smoking, *n* (%)	27 (34.18)	19 (31.15)	8 (44.44)	0.30
**Diabetes mellitus, ** ***n*** ** (%)**	**6 (7.59)**	**2 (3.28)**	**4 (22.22)**	**0.01**
Family history of CAD, *n* (%)	61 (77.22)	45 (73.77)	16 (88.88)	0.06

Notes: Data are represented by mean (M) ± standard deviation (m); median (Me) (25% and 75% quartile); frequency (percentage). Bold values signify statistical significance (*p* < 0.05). BMI—body mass index; CAD—coronary artery disease; NOCAD—nonobstructive coronary artery disease; OCAD—obstructive coronary artery disease.

**Table 2:** CT characteristics of NOCAD and OCAD patients.

Parameters	All Patients (*n* = 79)	NOCAD (*n* = 61)	OCAD (*n* = 18)	*p*-Value
Total Parameters of Coronary Atherosclerosis				
**TPV, mm^3^**	**114.00 ** **(52.80; 277.60)**	**88.90 ** **(40.40; 194.40)**	**278.60 ** **(179.50; 492.90)**	**<0.001**
**TPV-ST, mm^3^**	**4.60 (0.60; 20.10)**	**2.60 (0.00; 10.50)**	**30.50 (13.50; 67.50)**	**<0.001**
**TPV-FF, mm^3^**	**40.00 (15.60; 91.20)**	**31.30 (15.10; 73.00)**	**101.50 (68.90; 194.10)**	**<0.001**
**TPV-F, mm^3^**	**51.20 (21.00; 98.00)**	**43.00 (15.90; 78.20)**	**103.00 (53.40; 178.20)**	**<0.001**
**TPV-C, mm^3^**	**11.10 (0.00; 29.60)**	**9.50 (0.00; 26.50)**	**16.40 (9.00; 62.80)**	**0.032**
**TPV-NC, mm^3^**	**107.70 ** **(44.50; 221.70)**	**67.60 ** **(33.90; 165.00)**	**243.80 ** **(171.70; 360.80)**	**<0.001**
**TPB, %**	**4.75 (1.93; 9.95)**	**3.46 (1.65; 7.56)**	**11.27 (6.96; 16.68)**	**<0.001**
**TPB-ST, %**	**0.21 (0.03; 0.83)**	**0.11 (0.00; 0.41)**	**1.10 (0.69; 2.10)**	**<0.001**
**TPB-FF, %**	**1.75 (0.75; 4.01)**	**1.36 (0.44; 2.70)**	**4.10 (1.89; 9.76)**	**<0.001**
**TPB-F, %**	**2.14 (0.75; 4.16)**	**1.68 (0.67; 3.11)**	**4.72 (3.07; 6.13)**	**<0.001**
**TPB-C, %**	**0.46 (0.00; 1.37)**	**0.31 (0.00; 1.15)**	**1.04 (0.36; 2.98)**	**0.012**
**TPB-NC, %**	**4.16 (1.77; 9.00)**	**3.03 (1.29; 6.25)**	**9.45 (5.84; 15.52)**	**<0.001**
TLV, mm^3^	2345.40 (1807.80; 2861.70)	2383.30 (1977.20; 2861.70)	2055.25 (1599.30; 2836.80)	0.312
Parameters of PCAT				
PFAI (LAD), HU	−74.60 ± 6.93	−74.36 ± 6.62	−75.39 ± 8.05	0.057
PCAT volume (LAD), mm^3^	949.00 (664.00; 1180.00)	949.00 (734.00; 1180.00)	886.00 (593.00; 1111.00)	0.579
PFAI (LCX), HU	−67.47 ± 7.89	−66.84 ± 8.32	−69.60 ± 5.92	0.591
PCAT volume (LCX), mm^3^	376.00 (258.00; 635.00)	357.00 (270.00; 623.00)	425.50 (178.00; 803.00)	0.591
PFAI (RCA), HU	−74.45 ± 8.31	−73.51 ± 8.43	−77.61 ± 7.25	0.092
PCAT volume (RCA), mm^3^	1090.00 (698.00; 1530.00)	1120.00 (707.00; 1620.00)	978.00 (696.00; 1480.00)	0.788

Notes: TLV—total lumen volume; TPV—total plaque volume; TPV-ST—total plaque volume of the soft tissue component; TPV-FF—total plaque volume of the fibrous–fatty component; TPV-F—total plaque volume of the fibrous component; TPV-C—total plaque volume of the calcified component; TPV-NC—total plaque volume of the non-calcified component; TPB—total plaque burden; TPB-ST—total plaque burden of the soft tissue component; TPB-FF—total plaque burden of the fibrous–fatty component; TPB-F—total plaque burden of fibrous component; TPB-C—total plaque burden of the calcified component; TPB-NC—total plaque burden of the non-calcified component; PFAI—perivascular fat attenuation index; PCAT—pericoronary adipose tissue; LAD—left anterior descending artery; LCX—left circumflex artery; RCA—right coronary artery; HU—Hounsfield unit; OCAD—obstructive coronary artery disease; NOCAD—nonobstructive coronary artery disease. Bold values signify statistical significance (*p* < 0.05).

**Table 3:** Correlations between quantitative coronary atherosclerosis parameters and PCAT in vessels.

Parameters	PFAI (HU)	PCAT Volume (mm^3^)
Plaque volume (mm^3^)	−0.17	0.30
Plaque volume of soft tissue component (mm^3^)	−0.22	0.30
Plaque volume of fibrous–fatty component (mm^3^)	−0.17	0.27
Plaque volume of fibrous component (mm^3^)	−0.15	0.30
Plaque volume of calcified component (mm^3^)	NS	0.22
Plaque volume of non-calcified component (mm^3^)	−0.17	0.30
Plaque burden (%)	−0.14	0.18
Plaque burden of soft tissue component (%)	−0.21	0.26
Plaque burden of fibrous–fatty component (%)	−0.15	0.18
Plaque burden of fibrous component (%)	NS	0.18
Plaque burden of calcified component (%)	NS	0.19
Plaque burden of non-calcified component (%)	−0.14	0.18
Lumen volume (mm^3^)	−0.22	0.67

Notes. PFAI—perivascular fat attenuation index; PCAT—pericoronary adipose tissue; NS—not significant.

## Data Availability

The data presented in this study are available on request from the corresponding author. The data are not publicly available due to privacy considerations.

## Supplementary Materials

The following supporting information can be downloaded at: https://www.mdpi.com/article/10.3390/diagnostics16081174/s1, Table S1: Radiomic features with significant differences between NOCAD and OCAD patients.

## Abbreviations

The following abbreviations are used in this manuscript:

AMI	acute myocardial infarction
CAD	coronary artery disease
CCTA	coronary computed tomography angiography
CT	computed tomography
GFR	glomerular filtration rate
GLCM	gray-level co-occurrence matrix
GLDM	gray-level dependence matrix
GLRLM	gray-level run length matrix
GLSZM	gray-level size zone matrix
HU	Hounsfield unit
LAD	left anterior descending artery
LCX	left circumflex artery
MACE	major adverse cardiovascular event
NGTDM	neighborhood gray-tone difference matrix
NOCAD	nonobstructive coronary artery disease
OCAD	obstructive coronary artery disease
PCAT	pericoronary adipose tissue
PFAI	perivascular fat attenuation index
RCA	right coronary artery
