# Supplementary material for: Pericoronary Adipose Tissue Radiomic Features and Quantitative Plaque Analysis in Coronary Artery Disease: Insights from Coronary Computed Tomography Angiography
Source: Diagnostics (Basel). 2026 Apr 15;16(8):1174. doi: 10.3390/diagnostics16081174 (PMC13114650; doi:10.3390/diagnostics16081174)
Supplement: Supplementary file 1 [file diagnostics-16-01174-s001.zip › diagnostics-4206150-supplementary.pdf]

Supplementary Materials

Table S1. Radiomic Features with Significantly Differences between NOCAD and OCAD patients		
**Group**	**Feature**	**p-value**
**First-Order**	90Percentile	0.011
	Kurtosis	0.022
	Median	0.019
	Skewness	0.007
**GLCM**	Contrast	0.015
	Correlation	0.001
	DifferenceAverage	0.007
	DifferenceEntropy	0.012
	DifferenceVariance	0.030
	Id	0.003
	Idm	0.004
	Idmn	0.004
	Idn	0.002
	Imc1	<0.001
	Imc2	0.002
	MCC	0.001
	MaximumProbability	0.044
**GLDM**	DependenceNonUniformity	0.029
	DependenceNonUniformityNormalized	0.005
	DependenceVariance	0.005
	GrayLevelNonUniformity	0.002
	LargeDependenceEmphasis	0.003
	LargeDependenceHighGrayLevelEmphasis	0.002
	LargeDependenceLowGrayLevelEmphasis	0.015
	SmallDependenceEmphasis	0.002
	SmallDependenceHighGrayLevelEmphasis	0.016
	SmallDependenceLowGrayLevelEmphasis	0.004
**GLRLM**	GrayLevelNonUniformity	0.003
	LongRunEmphasis	0.002
	LongRunHighGrayLevelEmphasis	0.002
	RunLengthNonUniformity	0.003
	RunLengthNonUniformityNormalized	0.004
	RunPercentage	0.003
	RunVariance	0.001
	ShortRunEmphasis	0.004
**GLSZM**	LargeAreaEmphasis	0.008
	LargeAreaHighGrayLevelEmphasis	0.009
	LargeAreaLowGrayLevelEmphasis	0.010
	SizeZoneNonUniformityNormalized	<0.001

	SmallAreaEmphasis	0.001
	SmallAreaLowGrayLevelEmphasis	0.004
	ZoneEntropy	0.005
	ZonePercentage	0.003
	ZoneVariance	0.008
**NGTDM**	Busyness	0.019
	Coarseness	0.011

Table footnotes: GLCM – gray level co-occurrence matrix; GLDM – gray level dependence matrix; GLRLM – gray level run length matrix; GLSZM – gray level size zone matrix; NGTDM – neighborhood gray tone difference matrix; Id – inverse difference; Idm – inverse difference moment; Idmn – inverse difference moment normalized; Idn – inverse difference normalized; Imc1 – informational measure of correlation 1; Imc2 – informational measure of correlation 2; MCC – maximal correlation coefficient.
